# Primary ovarian insufficiency quality of life scale (POIQOLS): development and psychometric properties

**DOI:** 10.1186/s12905-022-02008-1

**Published:** 2022-11-28

**Authors:** Samira Golezar, Zohreh Keshavarz, Fahimeh Ramezani Tehrani, Abbas Ebadi, Farid Zayeri, Mohammad Hossein Golezar

**Affiliations:** 1 Department of Midwifery, Faculty of Nursing and Midwifery, Kermanshah University of Medical Sciences, Kermanshah, Iran; 2 Department of Midwifery and Reproductive Health, School of Nursing and Midwifery, Shahid Beheshti University of Medical Sciences, Tehran, Iran; 3 Reproductive Endocrinology Research Center, Research Institute for Endocrine Sciences, Shahid Beheshti University of Medical Sciences, Tehran, Iran; 4 Behavioral Sciences Research Center, Life Style Institute, Nursing Faculty of Baqiyatallah University of Medical Sciences, Tehran, Iran; 5 Department of Biostatistics, Proteomics Research Center, Faculty of Allied Medical Sciences, Shahid Beheshti University of Medical Sciences, Tehran, Iran; 6 Student Research Committee, Faculty of Medicine, Shahed University, Tehran, Iran

**Keywords:** Psychometric, Quality of life, Primary ovarian insufficiency, Scale

## Abstract

**Background:**

Primary ovarian insufficiency is menopause before the age of 40. It can affect the quality of life of afflicted women. Because there is no instrument available for measuring the quality of life of these women, the present study was carried out to develop and assess the psychometric properties of the quality of life scale for women with primary ovarian insufficiency.

**Methods:**

This exploratory sequential mixed method study was performed in two phases. In the qualitative phase (item generation), semi-structured in-depth interviews were conducted with 16 women having primary ovarian insufficiency, and a literature review was performed to generate initial items pool. In the quantitative phase (psychometric evaluation), the face, content, and construct validity (exploratory factor analysis), as well as reliability (internal consistency and test–retest methods), were evaluated. Besides, the responsiveness and interpretability were investigated.

**Results:**

During the first phase of the study, the initial item pool was generated with 132 items. After the face and content validity, the number of items was reduced to 40. The results of exploratory factor analysis yielded a 28 item scale with six factors. These factors explained 58.55% of the total variance. The Cronbach’s alpha for each factor was more than 0.7. Furthermore, the intraclass correlation coefficient for the entire scale was 0.95.

**Conclusions:**

The primary ovarian insufficiency quality of life scale (POIQOLS) is a valid and reliable tool for accessing the quality of life of women with primary ovarian insufficiency.

## Background

Primary ovarian insufficiency (POI) is menopause before the age of 40 [1]. The Study of Women’s Health Across the Nations showed that 1.1% of all women experience POI [2]. A meta-analysis revealed a 3.7% prevalence of POI among naturally menopausal women [3]. This disorder may occur spontaneously or as a result of medical interventions, including chemotherapy or bilateral oophorectomy [4]. Most of the cases occur sporadically and 3–4% of the POI occurrences have genetic and familial causes [5]. Women having POI are subject to psychosocial complications besides the physical effects of estrogen deficiency [6]. POI negatively affects the quality of life (QoL) and psychological health of afflicted women [7, 8]. high rate of depression and low self-esteem has been reported in these women due to the loss of fertility as well as the consequent sexual disorders [9]. Some studies have shown that POI negatively affects different aspects of Qol [10–12], thus it is necessary to evaluate the QoL of these women [13].

Nowadays, the QoL consequences of chronic diseases are taken into consideration [14]. Many people, including women with POI, suffer chronic diseases which diminish the quality of their lives. According to WHO, QoL is a multidimensional concept defined as an individuals’ perception of their position in life in the context of the culture and value systems in which they live concerning their goals, expectations, standards, and concerns [15]. In different studies, generic tools such as SF-36 (36-item, short-form survey) and the WHOQoL-BREF (World Health Organization Quality of Life Brief) have been used to assess the QoL of women with POI [10–12, 16].

The WHOQoL-BREF has four dimensions, physical, mental, environmental health, and social relations [17], while the SF36 concentrates on eight dimensions i.e. general health; physical performance; role restrictions owing to physical causes; role restrictions due to emotional causes; physical pain; social performance; energy and vitality; and mental health [18]. These generic tools are meant to apply to the general population, and that is why they ignore many aspects which can affect the QoL of women with POI and may not detect slight changes in the QoL of individuals suffering from different diseases [19]. A specific tool could thus be more appropriate to accurately assess the impact of POI on QoL. Specific QoL tools are sensitive towards changes in health care and could be used for critical treatment purposes and investigating the disease effects. Such tools provide us with invaluable data on issues affecting a person most and help in opting for the best health care plan [20].

To our knowledge, there is no specific tool to evaluate the QoL of women with POI; this served as the motivating factor for conducting the present study which aims at developing a QoL scale for women with POI and evaluating its psychometric properties.

## Methods

### Design and setting

This exploratory sequential mixed-methods study was conducted in two phases between July 2017 to November 2018. In the first phase, a qualitative study and a literature review were performed to generate the initial items pool. In the second phase, the psychometric properties of the scale were evaluated.

The study was conducted in the gynecology clinic of the Research Institute of Endocrine Sciences of Shahid Beheshti University of Medical Sciences, Tehran, Iran. The inclusion criteria were women with spontaneous POI, Iranian nationality, and not having a history of psychological or disabling chronic diseases.

### The first phase: item generation

In this study, the initial questionnaire was developed using the waltz 4-stage method [21]. To develop the items pool, the qualitative study (inductive approach) and literature review (deductive approach) were performed. The methods of the qualitative study have been reported in detail elsewhere [13]; in brief, the study population was 16 women with POI, who met the inclusion criteria. Purposive sampling was performed with a maximum variation of sampling which continued until data saturation. The semi-structured in-depth interviews were done by the main researcher (SG) in a private room. The interview duration varied between 40–105 min. The interviews began in July 2017 and ended in January 2018. The data were analyzed using a conventional content analysis approach following the method proposed by Graneheim & Lundman [22] and the themes and main categories were extracted. Also, to ensure the data rigor and conformability, the criteria proposed by Lincoln & Guba including credibility, dependability, conformability, and transferability were used [23].

Subsequently, a comprehensive literature review was performed using the keywords such as ‘Quality of Life’ AND ‘Questionnaire’ OR ‘Scale’ OR ‘Tool’ AND ‘POI’ OR ‘POF’ OR ‘Premature Menopause’ OR ‘Menopause’, in scientific databases such as Scopus, PubMed, Science Direct, Google Scholar, SID, and Magiran. The inclusion criteria were Persian or English sources, bearing the research keywords, and being published in accredited national/international scientific journals. The relevant articles published in the last thirty years were thoroughly studied and after comparing the items extracted from the qualitative study with those in the literature and removing duplicated items, the expert panel chose the most relevant ignored items to be added to the questionnaire in order to improve its comprehensiveness. As a result, the initial questionnaire was developed.

### The second phase of the study: psychometric evaluation

In this phase, the psychometric properties of POIQOLS were evaluated. validity of the questionnaire was assessed using face, content, and construct validity. Reliability was evaluated through internal consistency and test–retest methods. Besides, the responsiveness and interpretability of scale were investigated.

#### Face validity

To determine the face validity of the questionnaire, both qualitative and quantitative methods were used. To ensure qualitative face validity, 10 women with POI were asked to comment on the difficulty, irrelevancy, and ambiguity of the questionnaire items. To ensure quantitative face validity, the item-impact method was used. The 10 participants were asked to rate the importance of the items based on a 5-point Likert scale. Then, the impact score was calculated via multiplying the average of the importance rate of each item by the number of individuals who scored 4 or 5 on each item. An impact score greater than 1.5 is considered appropriate [24, 25].

#### Content validity

To determine the content validity of the questionnaire both qualitative and quantitative methods were used. To examine the qualitative content validity, the initial 90-item questionnaire was delivered to 10 experts in reproductive health, midwifery, and gynecology asking for their opinions on the grammar, wording, item placement, and scoring of the questionnaire items. The quantitative content validity was ensured using the content validity ratio (CVR) and content validity index (CVI).

To calculate the CVR, 10 experts were asked for their opinions on the essentiality of each item on a 3-point Likert scale. Then CVR was calculated using the following formula:$CVR = \frac{{nE - \frac{N}{2}}}{{\frac{N}{2}}}$ ($nE$= the number of experts who choose the necessary option, and N = total number of experts). The CVR cut-off point for 10 experts is 0.62 according to Lawshe Table [26]. To determine item‐level CVI (I-CVI), the relevancy of each item was calculated by dividing the number of experts putting a value of 3 or 4 values on an item by the total number of experts. An I-CVI value of ≥ 0.78 is acceptable. Then, modified Kappa statistics (k^*^) which adjusts the probability of chance agreement was calculated using the following formula: $P_{C} = \frac{{{\text{N}}!}}{{{\text{A}}!({\text{N}} - {\text{A}})!}} \times 0.5^{N} \to {\text{K}}^{*} = \frac{{{\text{I}} - {\text{CVI}} - {\text{Pc}}}}{{1 - {\text{Pc}}}}$ (P_C_: probability of chance agreement, N: the number of experts, A: the number of those agreeing on good relevance). A k^*^ value greater than 0.74 is excellent [27]. Finally, an average scale-level CVI (S-CVI/Ave) was evaluated. An S-CVI/Ave value of ≥ 0.8 is acceptable [28].

#### Item analysis

Before construct validity assessment, a pilot study on women with POI (n = 50) was conducted to evaluate internal consistency and identify poor items [29]. For this purpose, the corrected item correlation and the Loop method were used. Items with an item-total correlation coefficient of below 0.3 were removed. Also, items with an inter-item correlation coefficient of less than 0.3 and greater than 0.8 were omitted. The loop method is Cronbach’s Alpha if the item is deleted, and the values increase for poor and inappropriate items [30].

#### Construct validity

In this study, the construct validity was evaluated using exploratory factor analysis. Different resources consider 3–10 participants per item as appropriate. Another viewpoint regarding the sample size is that 100 to 200 participants suffice as long as the correlation is measured [31]. Therefore, 120 women with POI who met the inclusion criteria were chosen conveniently. They were asked to complete the questionnaire via self-administration electronically. The Kaiser-Maier-Olkin (KMO) statistic of sampling adequacy and Bartlett’s test were calculated to check the appropriateness of the data for factor analysis. A KMO value of 0.8 was considered acceptable [31]. To determine the number of extractable factors, eigenvalue greater than 1 and scree plot were used. Also, the minimum acceptable factor loading value was determined as 0.47 based on the following formula: CV = 5.152 ÷ √ (n –2) [32].

#### Reliability and responsiveness

To ensure the reliability of the scale, internal consistency, and test–retest method were used. The internal consistency was assessed using the final questionnaire after construct validity with the same samples of the construct validity using Cronbach’s alpha coefficient. A value greater than 0.7 was regarded as acceptable [33]. The test–retest method using the inter-class correlation coefficient (ICC) was conducted to investigate relative stability. Doing this, 30 women with POI were requested to fill the questionnaire twice at a two-week interval. An ICC higher than 0.8 indicated satisfactory stability [30].

To determine responsiveness, standard error of measurement (SEM) and the minimal detectable change (MDC) score were calculated [34]. The SEM formula is given by: SEM = SD $\surd 1$ – ICC; Where SD is pooled standard deviation of the test and the retest [35]. The lower the SEM, the higher the reliability will be [25]. MDC is a real change that doesn't come from measurement error [36]. The MDC formula is given by: MDC = SEM × √2 × 1.96. The MDC less than 30% is acceptable, and below 10% is regarded as excellent [36].

#### Interpretability

To determine the interpretability, the distribution of total scores in the whole samples, and the ceiling and floor effects were calculated [37]. The ceiling and floor effects are assumed to exist when more than 20% of the respondents obtain the highest or lowest achievable score of the scale [30].

#### Statistical analysis

All data were analyzed using the SPSS-AMOS (v.22). Univariate normality was assessed using skewness (± 3) and kurtosis (± 7). Multivariate normality was assessed via determinant (*p* > 0.0001), and multivariate outliers were assessed by the Mahalanobis d-squared (*p* < 0.001) [38]. The latent factors were extracted using the maximum-likelihood EFA with a Varimax Rotation. Missing data were assessed via multiple imputations and were replaced with the mean of participants’ scores. Cronbach’s coefficient alpha and ICC were also calculated. An independent t-test was used to compare the mean scores of QoL in the two groups. *p* values < 0.05 were considered as significant.

## Results

### Item generation

In the Qualitative study, 16 women with POI aged 28–47 years old, with a disease duration of 2–15 years were interviewed. 10 of the participants were married, 9 of them were employed, 6 had children (2 donor oocyte recipients), 7 had a family POI history, and they had various education levels. After content analysis of the interviews focusing on the concepts of the QoL of women with POI, 5 themes of disease effects, distorted self-concept, fears & concerns, hormone replacement therapy (HRT) effects, and coping strategies were extracted [8, 13].

Based on the deductive-inductive questionnaire development approach, the initial items pool was developed which consisted of 132 items. 128 items related to the qualitative study and 4 items related to the literature review. The items pool was thoroughly refined in several rounds to remove the repetition or overlap of items. Ultimately, 90 items remained in the items pool including disease effects (21 items); distorted self-concept (15 items); fears & concerns (16 items); HRT effects (13 items); and coping strategies (25 items).

### Face and content validity

The results of qualitative face validity showed that nine items needed to be modified. Also, twelve items with an impact score of smaller than 1.5 were revised in the quantitative face validity evaluation.

Sixteen items were modified during the qualitative content validity assessment. After setting the cut-off point for the quantitative validity indices i.e. CVR > 0.62; CVI > 0.79, and k* > 0.74, the number of items was reduced to 50. Also, the S-CVI/Ave of the 50-item POIQOLS was calculated as 0.96.

### Item analysis

In this stage, the 50-item questionnaire was administered to 50 women with POI. The scale-level Cronbach's alpha coefficient was calculated as 0.89. Considering item-total and inter-item correlations of less than 0.30, ten items were eliminated. Also, the inter-item correlation coefficient for all items was lower than 0.8.

### Construct validity

For exploratory factor analysis, the 40-item scale resulting from the item analysis was completed by 120 women with POI aged 36.21 ± 6.53 years with a disease duration of 7.30 ± 9.54 years. A detailed account of the demographic and reproductive characteristics of the participants could be found in Table 1.

**Table 1:** Demographic and reproductive characteristics of participants (N = 120)

Characteristics	Mean ± SD
Age (year)	36.21 ± 6.53
Menarche age (year)	13.55 ± 1.74
Disease duration (year)	7.30 ± 9.54
*Characteristics*	N (%)
Education level	
Elementary	12 (10)
High school/diploma	37 (30.8)
University degree	71 (59.2)
Occupation	
Employed	47 (39.2)
Unemployed	73 (60.8)
Income level	
Sufficient	31 (25.8)
Moderately sufficient	68 (56.7)
Insufficient	21 (17.5)
Marital status	
Single	20 (16.7)
Married	100 (83.3)
Number of children	
0	46 (38.3)
≥ 1	74 (61.7)

**Fig. 1:**
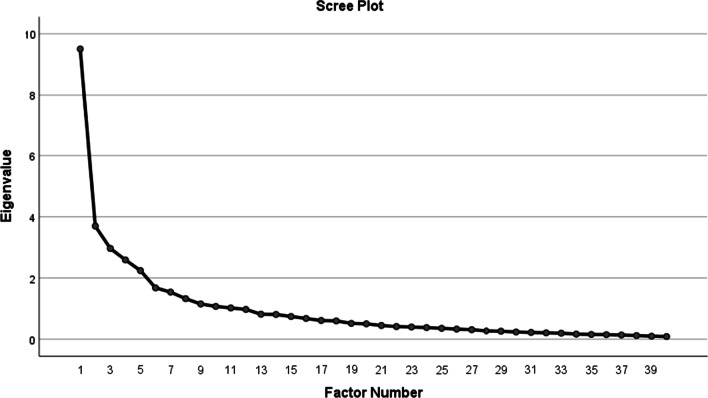
Determining the number of factors constructing the POIQOLS

The KMO test value was 0.81, and Bartlett's test was significant (χ^2^ = 1836/522, *p* < 0.001). A maximum likelihood EFA with Varimax rotation was performed after restricting the number of factors to 6 based on the Eigenvalues greater than 1 and scree plot (Fig. 1). The eigenvalues of these 6 factors were 3.14, 2.99, 2.92, 2.80, 2.68, and 1.83 respectively; they explained 58.55% of the total variance of the scale (Table 2). The items were subsumed under the factor with the highest factor load. 12 items that were not loaded on any of the factors with a factor load of less than 0.47 were excluded from the scale whereby 28 items remained. Subsequently, each factor was labeled according to its items. The 6 factors in POIQOLS included psychological effects (5 items), coping strategies (6 items), HRT complications (4 items), fears & concerns (6 items), self-concept (4 items), and sexual function (3 items).

**Table 2:** The explained variances and eigenvalues of the POIQOLS dimensions and the factor loadings and the communality values of their items

Factor	Items	Factor Loadings	Communality	Eigenvalue	Variance (%)
Psychological effects	8. I have got sensitive and irritable	0.77	0.71	3.14	11.23
	7. I have become depressed and introverted	0.73	0.70		
	9. I have stress and anxiety	0.71	0.61		
	6. I have become nervous and aggressive	0.70	0.57		
	5. I feel sad due to some complications of the disease (weight gain, sexual problems, infertility, and osteoporosis)	0.53	0.49		
Coping strategies	31. It is easier for me to tolerate the disease when I am engaged in various task types	0.84	0.74	2.99	10.68
	32. It is easier for me to tolerate the disease when I think positively and instill positive thinking	0.80	0.69		
	35. It is easier for me to tolerate the disease after I have gained valid information from different sources	0.60	0.44		
	34. It is easier for me to tolerate the disease after I have removed getting married or having children as my goals in life	0.58	0.46		
	33. Prayers and trust in God have helped me tolerate the disease	0.56	0.43		
	30. The pass of the time has helped me tolerate the disease easier	0.50	0.32		
HRT complications	26. I am tired of prolonged daily use of hormonal medicine	0.83	0.73	2.92	10.45
	27. Hormone therapy has caused me to gain weight and has messed with my fitness	0.80	0.68		
	28. Hormone therapy has caused my hair to fall and get thin	0.76	0.65		
	29. Hormone therapy has caused blemishes on my face	0.69	0.63		
Fears & concerns	23. I am afraid one day the egg donor might someday come after my child	0.70	0.54	2.80	10.01
	21. I am afraid I might not get cured	0.65	0.61		
	24. I am afraid others might know about my problem	0.59	0.60		
	22. I am afraid I might not be able to have a child	0.59	0.60		
	25. I am afraid I might become lonely	0.58	0.53		
	19. I am afraid one day my daughter might have POI	0.57	0.40		
Self-concept	15. I have lost my self-confidence	0.74	0.73	2.68	9.60
	16. I feel I am not a perfect woman	0.70	0.64		
	14. I feel old and withered	0.66	0.64		
	17. I am annoyed with being labeled as menopausal and being compared to old women	0.64	0.49		
Sexual function	12. My intercourse with my husband is out of obligation	0.78	0.63	1.83	6.56
	11. My sexual desire is reduced	0.77	0.65		
	10. I have pain during vaginal penetration	0.53	0.38		

### Reliability and responsiveness

Internal consistency assessment revealed that Cronbach's alpha values of POIQOLS sub-scales ranged from 0.76 to 0.88. The relative stability was assessed by the test–retest method via ICC which was calculated as 0.95 (CI:0.90–0.97, p < 0.001) for the entire scale and ranged from 0.85 to 0.95 for sub-scales (Table 3). The SEM and MDC were ± 5.69 and 10.24, respectively. MDC percentage was calculated as less than ten percent (6.31).

**Table 3:** Internal consistency and relative stability of POIQOLS

Factor	Cronbach’s alpha	ICC (95% confidence interval)
Psychologic	0.87	0.92 (0.84–0.96)
Coping strategies	0. 81	0.91 (0.83–0.95)
HRT complications	0.88	0.95 (0.91–0.97)
Fear and concern	0.83	0.90 (0.81–0.95)
Self-concept	0.84	0.89 (0.79–0.94)
Sexual function	0.76	0.85 (0.71–0.92)

### Interpretability

The results of calculating the ceiling and floor effects showed that the percentage of minimum and the maximum score for the entire scale and six sub-scales were below 20%. In addition, the mean score of QoL was different in women with different marital and employment statuses (Table 4).

**Table 4:** Distribution of QoL scores in participants (n = 120)

Variable	Mean ± SD	Result
*Marital status*		
Single	93.15 ± 14.97	F = 3.2, df = 118; p = 0.003
Married	78.80 ± 19.65	
*Occupation*		
Employed	87.70 ± 20.17	F = 0.16, df = 118; p = 0.003
Unemployed	76.93 ± 18.18	

### Scoring rules

The final POIQOLS consists of 28 items in 6 domains. Each item was rated on a five-point Likert scale ranging from strongly agree to strongly disagree; always to never; very much to not at all and scored from 1 to 5. The items relating to the sub-scale ‘coping strategies’ were reverse-scored (from 5 to 1). The total score of the scale ranged from 28 to 140. The higher the obtained score, the higher the QoL would be. The scores of these items for women who had not taken HRT and also those who did not have a sexual partner were considered as 3 (to some extent and sometimes, respectively). Then, the total scores of POIQOLS and its dimensions were transformed to standard score (0 to 100), via the linear method using the following conversion formula where a higher score is indicative of a higher level of QoL.

$$Transformed \, Score = \frac{{\left( {Actual \, raw \, score - Lowest \, possible \, raw \, score} \right)}}{{\left( {Highest \, possible \, raw \, score - Lowest \, possible \, raw \, score} \right)}} \times 100$$

## Discussion

The aim of this study was to develop and evaluate the psychometric properties of POIQOLS. The results of the study suggested that the scale has acceptable validity and reliability. POIQOLS included 28 items and six subscales consisting of psychological effects, coping strategies, HRT complications, fears & concerns, self-conception, and sexual function which explained 58.55% of the total variance. The acceptable explained variance of the scale confirms its ability to measure the concept of QoL among women with POI.

The first subscale extracted in the EFA was the 'psychological effects’, which included 5 items relating to the psychological effects of POI. This factor with the highest percentage of variance is considered as one of the critical dimensions of this tool. Studies have shown that POI has many psychological problems for women [6] which negatively affects their QoL [7]. POI is a type of infertility that occurs due to the loss of normal ovarian function before the age of 40. Therefore, the consequences of such a diagnosis can be emotionally and psychologically devastating [39]. Women with POI have reported having feelings such as grief, depression, anxiety, and emotional distress [40–42].

The second subscale, which was reverse-scored, was ‘coping strategies’. It included 6 items relating to adaptation to POI. There is so much variation in adjustment to POI and many women with the condition adapt well [39]. The results of a study showed that women with POI tried to ignore the disorder via living for the moment and entertaining themselves [43]. Contrary to this, an investigation of infertile women with POI showed that strategies like avoidance and letting go/moving on could help infertile women in the short term but may not turn out to be advantageous in the long run and make distress last longer in these women [39]. The studies have shown that the type of attitude toward the disease affects QoL. Optimism and positive beliefs affect a person’s physical and psychological health and help them to cope with stressful occasions [40, 44].

Besides a feeling of purposiveness, the ability to problem-solving, and believe in a bright future have been mentioned as the characteristics of resilient persons which enhance psychological health [45]. Infertile women have been reported as having better adjustment abilities if they manage to rearrange their goals, forget about their previous goals, and reassess themselves positively [39]. A study showed that substituting childbearing with other goals and getting engaged with the new goals affects women with POI positively and helps them get themselves together after a period of difficulty [46]. Results of several studies suggest that religion and spirituality could decrease stress in these women [9, 54]. One of the important factors for coping with POI is providing accurate information about the disorder by clinicians [8, 41, 46].

The third subscale of the tool was the 'HRT complications' which included 4 items. The items of this dimension are related to the physical and psychological effects of HRT. It is recommended that in women with POI, HRT be continued until the age of natural menopause (51yrs) on the condition that no contraindication is present [9, 47]. Some studies have shown that HRT could lead to the improvement of QoL in menopausal women [48]. Singer et.al. indicated that long-term consumption of HRT is one of the difficulties of women with POI [12]. Weight gain is one of the unpleasant side effects of menopausal women taking HRT [49]. Moreover, the consumption of oral contraceptives is related to hair loss and pigmented patches of skin [50, 51]. These side effects could distort self-image in women with POI and reduce QoL.

The fourth subscale in the present study was 'fears & concerns' that included 6 items. These items included fears and concerns related to health issues, such as fear of infertility, no remedy, and inherited POI, as well as those related to distorted self-concepts such as fear of loneliness and POI disclosure. In recent studies, women with POI have reported concern and anxiety frequently [40–42]. Boughton reported that women with POI were worried about others knowing about their menopausal status [52]. Another study reported higher levels of anxiety and depressive symptoms in patients who felt more stigmatization and uncertainty with regards to their disorder [46].

The fifth subscale of POIQOLS was 'self-concept' which included 4 items relating to the attitude of women toward their disorder. Attitude toward oneself is called ‘self-concept’ [40] which covers all the positive and negative aspects of a person [53]. Health-related conditions such as the psycho-social ones, sadness, and loss, cause the self-concept to change. Moreover, self-concept and QoL are directly related [54]. A diagnosis of POI in young women has been associated with feelings of loss, guilt, shame, oldness, low self-esteem, less femininity, and negative feelings regarding their fertility [41]. Besides, infertile women may face rumination, catastrophizing, and self-blame that are accompanied by maladaptive outcomes, such as depression, negative effects, and infertility-related distress [39].

The last subscale of the POIQOLS was 'sexual function', which included 3 items relating to perceived sexual coercion, a decrease in sexual desire, and painful intercourse. Sexual dysfunction negatively affects QoL [55]. POI affects sexual function and sexual relations via POI-induced psychic trauma, affecting sexual desire, sexual arousal, orgasm, vaginal receptivity, partner-related issues, and infertility concerns [47]. A study reported that women who have failed to bear at least one child before POI are prone to losing their motivation to initiate or be open to having intercourse [56]. Also, many studies have reported POI women as experiencing loss of sexual desire, vaginal dryness, and dyspareunia [12, 42, 57].

The Cronbach’s alpha coefficient of all dimensions of POIQOLS showed the acceptable internal consistency of the scale. Moreover, test–retest ICC values revealed that the scale has acceptable stability. The result of MDC percent showed that the POIQOLS has excellent responsiveness. Also, the distribution of QoL scores in the samples and the lack of ceiling and floor effects indicated the interpretability of the scale.

The strength of this study is the development of a specific tool to assess the QoL of women with POI based on empirical data and existing literature and evaluate its validity through robust methods. Among the limitations of our study was that women with POI were hard to reach. To cope with this problem, an electronic questionnaire was developed and the participants were provided with a contact number for consultation purposes and to try and increase their cooperation. Also, this questionnaire was developed in Iran and may reflect only the language and culture of the Iranian society, Therefore, it is recommended that psychometric evaluation of this scale be assessed with different cultures and communities.

## Conclusion

The POIQOLS has good validity and reliability that can be used to help healthcare providers to assess the QoL of women with POI, take appropriate measures to improve their QoL, and evaluate the impact of the services provided. Also, using this scale can provide effective information to health system managers and decision-makers for resource allocation. It seems that due to its good psychometric properties as well as its ease of administration and applicability, the POIQOLS can fill the gap of a QoL questionnaire for women with POI.

## Data Availability

The datasets generated and/or analysed during the current study are not publicly available due to privacy and confidentiality agreements as well as other restrictions, but are available from the corresponding author on reasonable request.

## Abbreviations

POI: Primary ovarian insufficiency
QoL: Quality of life
HRT: Hormone replacement therapy
CVI: Content validity index
CVR: Content validity ratio
SEM: Standard error measurement
I-CVI: Item‐level content validity index
S-CVI: Scale-level Content Validity Index
S-CVI/Ave: Scale-level Content Validity Index average
MDC: Minimal detectable changes
KMO: Kaiser–Maier–Olkin
SD: Standard deviation
SF-36: 36-Item Short-Form Health Survey
WHOQOL-BREF: World Health Organization quality of life assessment
MENQOL: Menopause-specific quality of life questionnaire
MRQ: Menopause representations questionnaire
MSSI-38: Menopause symptoms severity inventory
MRS: Menopause rating scale
